# Association of Psychoactive Substance Use With Syphilis and HIV Positivity Among Pregnant Women Receiving Prenatal Care in Belém, Northern Brazil

**DOI:** 10.1155/jotm/3435193

**Published:** 2026-09-03

**Authors:** Luciano Chaves Franco Filho, Herald Souza dos Reis, Yan Corrêa Rodrigues, Cintya de Oliveira Souza, Felipe Bomfim Freitas, Rodrigo Santos de Oliveira, Gabriel Silas Marinho Sousa, Maria Luiza Bazzo, Pamela Cristina Gaspar, Adele Schwartz Benzaken, Joana da Felicidade Ribeiro Favacho, Daniela Cristina Soares Vieira da Silva

**Affiliations:** ^1^ Instituto Evandro Chagas, Ananindeua, Pará, Brazil, iec.gov.br; ^2^ Microbiology Laboratory, Serra Talhada Academic Unit (UAST), Federal Rural University of Pernambuco (UFRPE), Av. Gregório Ferraz Nogueira, Serra Talhada 56909-535, State of Pernambuco, Brazil, ufrpe.br; ^3^ Centro de Ciências da Sáude, Universidade Federal de Santa Catarina, Florianópolis, State of Santa Catarina, Brazil, ufsc.br; ^4^ Ministry of Health Department of HIV and AIDS, Brasilia 70723-040, Brazil; ^5^ Instituto Leonidas e Maria Deane Fiocruz Amazonia., Manaus, State of Amazonas, Brazil

## Abstract

Vertical transmission of sexually transmitted infections remains a public health concern, particularly in socially vulnerable settings. Although Brazil has achieved the elimination of mother‐to‐child transmission of HIV, syphilis during pregnancy continues to pose significant challenges, especially in the northern region. This cross‐sectional study aimed to estimate the prevalence of HIV and syphilis and identify associated factors among pregnant women receiving prenatal care in Belém, Pará, Brazil. Between April and June 2021, 398 pregnant women were interviewed using a structured clinical–epidemiological questionnaire and underwent laboratory testing. HIV diagnosis was established using fourth‐generation serological assays with immunoblot confirmation, and syphilis diagnosis was based on nontreponemal testing followed by treponemal confirmation. Descriptive analyses were performed, followed by bivariate analyses using Fisher’s exact test, chi‐square test with Monte Carlo simulation, and Mann–Whitney U test, and Poisson regression with robust variance was applied to estimate prevalence ratios for syphilis. The prevalence of syphilis was 6.36%, and HIV prevalence was 1.01%. Psychoactive substance use was the only variable independently associated with syphilis positivity, while other sociodemographic and behavioral factors showed descriptive trends without statistical significance, and no significant associations were observed for HIV infection. These findings indicate that persistent social and behavioral vulnerabilities, particularly psychoactive substance use, contribute to ongoing syphilis transmission during pregnancy, underscoring the importance of integrating substance use screening, targeted counseling, and harm‐reduction strategies into prenatal care to advance efforts toward eliminating vertical transmission of sexually transmitted infections.

## 1. Introduction

In Brazil, the elimination of vertical transmission of sexually transmitted infections is a central goal of public health policies and aligns with the United Nations Sustainable Development Goals (SDGs). Brazil has already achieved the elimination of mother‐to‐child transmission of HIV, as validated by international health authorities, representing a major milestone in national HIV and AIDS control efforts [1]. This achievement directly contributes to SDG 3 (Good Health and Well‐being), particularly Targets 3.1 and 3.2, which aim to reduce maternal and neonatal mortality, and Target 3.3, which seeks to end the AIDS epidemic. Furthermore, these advances reinforce SDG 5 (gender equality) by promoting universal access to sexual and reproductive health services (Target 5.6). Despite this progress, the elimination of vertical transmission of syphilis remains an ongoing challenge, underscoring the need to strengthen prenatal screening, timely diagnosis, and adequate treatment to achieve comprehensive elimination by 2030. Vertical transmission can have severe consequences for both the newborn and the mother, including miscarriages, maternal or neonatal deaths, and serious child health complications [2]. These impacts underscore the urgency of effective interventions that integrate early diagnosis, appropriate treatment, and health education to prevent new cases.

The Brazilian Ministry of Health provides a range of resources to municipalities, including rapid tests, medications, online training courses for healthcare professionals, and specific guidelines to ensure diagnosis and treatment are conducted at the patient’s point of care. However, case notification rates remain alarming, indicating that additional barriers, such as social and structural factors, directly hinder the achievement of certification for the elimination of vertical transmission of syphilis by 2030 [3].

Since 2020, Brazil has made substantial progress in preventing mother‐to‐child transmission of HIV, despite the disruptions caused by the COVID‐19 pandemic. In 2024, the national detection rate of HIV infection among pregnant women was 3.2 cases per 1000 live births, remaining relatively stable in recent years [3]. In parallel, surveillance data indicate a continued reduction in AIDS incidence among children under 5 years of age, reflecting the effectiveness of prenatal screening, early diagnosis, and antiretroviral therapy [3]. These results supported Brazil’s international recognition for achieving the elimination of vertical transmission of HIV as a public health problem [1]. In contrast, syphilis in pregnancy remains a major challenge, with 35.4 cases per 1000 live births reported among pregnant women in 2024 at the national level. Although a decline in congenital syphilis has been observed since 2022, persistent regional and social inequalities, delays in diagnosis, inadequate treatment of pregnant women and their partners, and gaps in prenatal follow‐up continue to sustain vertical transmission, underscoring the need to strengthen surveillance and maternal and child health strategies to advance toward the elimination of congenital syphilis [4].

In the State of Pará, surveillance data indicate that in 2024, the detection rate of HIV infection among pregnant women reached 4.2 cases per 1000 live births, exceeding the national rate of 3.2 per 1000 live births [3]. Although prenatal care coverage among pregnant women living with HIV remains high, the reported use of antiretroviral therapy during pregnancy was 72.4% in 2024, below the target required for the elimination of vertical transmission. Regarding syphilis, national data show a detection rate of 35.4 cases per 1000 live births among pregnant women, with Pará remaining among the states with elevated detection rates. Despite recent national reductions in congenital syphilis, vertical transmission of syphilis continues to pose a significant public health challenge in the state [4].

Belém, the capital of the state of Pará, is in the northern region of Brazil, along the Guajará Bay, with geographic coordinates of latitude 1°27′21″S and longitude 48°30′14″W. It is one of the main urban centers in Amazon and plays a strategic role in the region’s economy and culture. Despite its significance, the city faces significant challenges related to precarious infrastructure and the living conditions of its population. Belém exhibits high levels of social inequality, elevated rates of violent deaths, difficulties in accessing healthcare services, particularly in the more peripheral and insular areas, and social vulnerabilities associated with psychoactive substance use, all of which may contribute to adverse health outcomes and increased exposure to infectious diseases [5].

In this study, we conducted interviews and diagnostic testing with pregnant women receiving prenatal care at health units in the municipality of Belém between April and June 2021. The objective was to assess the prevalence of syphilis and HIV among pregnant women and to identify the main factors hindering the interruption of vertical transmission of these infections. The findings aim to provide evidence to support integrated strategies that address social inequalities, strengthen access to healthcare services, and contribute to achieving the targets established in the SDGs, thereby improving maternal and child health outcomes.

## 2. Materials and Methods

### 2.1. Study Design

This study employed a cross‐sectional design, conducted to estimate the prevalence of syphilis and HIV among pregnant women and to identify factors associated with infection and barriers to the interruption of vertical transmission.

### 2.2. Setting

The city of Belém (state of Pará, Brazil) has an estimated population of 1.3 million inhabitants [6]. Its territory is predominantly insular, consisting of 42 islands, which represent 65% of its total area. The municipal administration is organized into eight administrative districts encompassing 71 neighborhoods. The municipality has 31 Basic Health Units (UBS), which provide essential services, such as prenatal care for pregnant women. The study was carried out between April and June 2021 in three Basic Health Units (neighborhoods of Icoaraci, Bengui II, and Paraíso dos Pássaros) located in the municipality of Belém. These units are part of the public primary healthcare network and provide routine prenatal care. Data collection occurred during scheduled morning and afternoon visits during the study period.

### 2.3. Inclusion and Exclusion Criteria

This study included individuals aged 18 years or older who identified as women and were undergoing prenatal care. Exclusion criteria comprised individuals who did not provide informed consent, those under the age of 18, and individuals under the influence of alcohol or drugs at the time of participation.

### 2.4. Variables

The primary outcomes were laboratory‐confirmed syphilis and HIV infection. Explanatory variables included sociodemographic characteristics (age, self‐reported skin color, education level, household income, and marital status), behavioral factors (age at sexual initiation, condom use, number of sexual partners, alcohol consumption, and psychoactive substance use [oxi, cocaine paste, LSD, cannabis, and cocaine]), and access to healthcare indicators (timing of prenatal care initiation, previous testing for HIV and syphilis, and knowledge of mother‐to‐child transmission).

### 2.5. Data Sources and Measurement

Data were collected using a structured clinical–epidemiological questionnaire administered by trained personnel. HIV diagnosis was established using fourth‐generation serological assays, with confirmatory testing by immunoblot, considered the reference standard. Syphilis diagnosis was based on nontreponemal testing (VDRL) followed by confirmation with treponemal assays (FTA‐ABS), in accordance with Brazilian Ministry of Health guidelines [7, 8]. All laboratory analyses were performed by the technical team of the Evandro Chagas Institute, ensuring standardized diagnostic procedures across participants.

### 2.6. Study Size

The minimum sample size was calculated using the formula *n* = (*Z* ^2^ × *P* × (1 − *P*))/*e* ^2^, where *Z* corresponds to the standard normal deviate for a 95% confidence level (*Z* = 1.96), *P* represents the expected prevalence, and *e* denotes the margin of error set at 3%. Assuming an expected prevalence of 5% [9], the minimum required sample size was estimated at 203 pregnant women.

### 2.7. Statistical Methods

Descriptive analyses were conducted to characterize the study population. Bivariate analyses were performed using Fisher’s exact test for binary variables, the chi‐square test with Monte Carlo simulation for categorical variables with more than two categories, and the Mann–Whitney U test for numerical variables. To estimate adjusted associations, a Poisson regression model with robust variance was applied to calculate prevalence ratios and 95% confidence intervals. Missing data were assessed during database curation and were minimal; therefore, complete‐case analysis was performed. No subgroup or sensitivity analyses were conducted.

### 2.8. Ethical Considerations

The present study was approved by the Research Ethics Committee of the Evandro Chagas Institute (No. 19146919.3.0000.0019; approval date: August 27, 2019) and the Pan American Health Organization (No. PAHOERC‐2019‐08‐0059; approval date: August 27, 2019). Authorization was also obtained from the Permanent Education Center of the Health Department of Belém (July 25, 2019). Participants were recruited during routine prenatal care visits at the participating healthcare units and were individually invited to participate in the study. Participation was entirely voluntary, and all women provided written informed consent before enrollment. Refusal to participate did not interfere with access to prenatal care or any other healthcare services. Study data and blood samples were identified using unique identification codes to ensure participant confidentiality and data protection.

## 3. Results

Psychoactive substance use was the only variable significantly associated with syphilis positivity (*p* < 0.05) (Table 1). Other sociodemographic and behavioral variables, including household income, age at sexual debut, educational level, condom use, prior knowledge of infections, and previous testing, showed no statistically significant associations in the final analysis. No statistically significant associations were identified for HIV infection.

**TABLE 1:** Bivariate and Poisson regression analyses of factors associated with HIV and syphilis among pregnant women in Belém, Brazil.

**Variable**	**Variable type**	**Model**	***p*-value (HIV)**		***p*-value (syphilis)**

Age (years)	Numeric	Mann–Whitney U	0.881		0.976
Age group	Categorical	Chi‐square (Monte Carlo)	0.964		0.613
Race/skin color	Categorical	Chi‐square (Monte Carlo)	0.891		0.734
Education level	Categorical	Chi‐square (Monte Carlo)	0.917		0.698
Household income	Categorical	Chi‐square (Monte Carlo)	0.903		0.721
Marital status	Categorical	Chi‐square (Monte Carlo)	0.955		0.756
Sexual orientation	Categorical	Chi‐square (Monte Carlo)	0.889		0.709
Knowledge about HIV	Binary	Fisher’s exact test	1		1
Knowledge about syphilis	Binary	Fisher’s exact test	0.446		1
Previous HIV testing	Binary	Fisher’s exact test	0.812		0.887
Previous syphilis testing	Binary	Fisher’s exact test	0.901		0.904
Alcohol consumption	Binary	Fisher’s exact test	0.301		1
Psychoactive substance use	Binary	Fisher’s exact test	0.401		0.019
Condom use (regular partner)	Categorical	Chi‐square (Monte Carlo)	0.941		0.668
Condom use (casual partner)	Categorical	Chi‐square (Monte Carlo)	0.926		0.651
Regular sexual partner	Binary	Fisher’s exact test	0.784		0.742
Casual sexual partner	Binary	Fisher’s exact test	0.821		0.713
Number of casual partners	Categorical	Chi‐square (Monte Carlo)	0.742		0.681

**Variable**	**Model**	**Prevalence ratio (PR)**	**95% CI (lower)**	**95% CI (upper)**	***p*-value**

Psychoactive substance (yes vs no)	Poisson regression with robust variance	2.9	1.28	6.56	0.0109

*Note:* Bivariate analyses used Fisher’s exact test, chi‐square test with Monte Carlo simulation, and Mann–Whitney U test, as appropriate. Adjusted associations with syphilis are presented as prevalence ratios (PR) with 95% confidence intervals.

Among the 398 pregnant women evaluated, 25 tested positive for syphilis, representing a prevalence of 6.36% (95% CI: 4.12%–9.16%) (Table 2). Pregnant women with positive syphilis results were not clinically evaluated as part of this study. Four women tested positive for HIV, corresponding to a prevalence of 1.01% (95% CI: 0.28%–2.56%) (Table 2). Of these, two had never previously tested positive for HIV, one was aware of her HIV‐positive status and was receiving regular treatment, and one preferred not to disclose whether she had undergone previous HIV testing or was aware of her serological status. Among the women tested, the majority identified as “Brown skin” (67.34%), followed by “Black skin” (14.32%) and “White skin” (12.06%). The prevalence of positive results for HIV (100%) and syphilis (81.66%) was higher among Black‐ and Brown‐skinned women (Table 3).

**TABLE 2:** Numbers of positive and negative cases and prevalence values of the evaluated samples (reference tests).

Pregnant women	Syphilis	HIV
Positive	25	4
Negative	373	394
Prevalence	6.36% (4.12%–9.16%)	1.01% (0.28%–2.56%)
Total	**398**	**398**

*Note:* The bold values refer to total patients evaluated.

**TABLE 3:** Distribution of sociodemographic and socioeconomic characteristics according to syphilis and HIV status.

		Syphilis					HIV				
	Total	Positive	Negative	Total (%)	Positive (%)	Negative (%)	Positive	Negative	Total (%)	Positive (%)	Negative (%)
*Ethnicity*											
White skin	48	3	45	12.09	12.00	12.10	0	48	12.09	0.00	12.21
Black skin	57	5	52	14.36	20.00	13.98	1	56	14.36	25.00	14.25
Yellow skin	19	2	17	4.79	8.00	4.57	0	19	4.79	0.00	4.83
Brown skin	267	15	252	67.25	60.00	67.74	3	264	67.25	75.00	67.18
Indigenous	6	0	6	1.51	0.00	1.61	0	6	1.51	0.00	1.53
Total	397	25	372	100.00	100.00	100.00	4	393	100.00	100.00	100.00

*Education*											
Incomplete elementary (1–3 years of education)	1	1	0	0.25	4.00	0.00	0	1	0.25	0.00	0.25
Incomplete fundamental	46	4	42	11.59	16.00	11.29	1	45	11.59	25.00	11.45
Incomplete high school	94	13	81	23.68	52.00	21.77	1	93	23.68	25.00	23.66
Completed high school	204	6	198	51.39	24.00	53.23	2	202	51.39	50.00	51.40
Incomplete higher education	30	1	29	7.56	4.00	7.80	0	30	7.56	0.00	7.63
Completed higher education or more	22	0	22	5.54	0.00	5.91	0	22	5.54	0.00	5.60
Total	397	25	372	100.00	100.00	100.00	4	393	100.00	100.00	100.00

*Monthly family income*											
R$1.00 to R$500.00	39	7	32	9.82	28.00	8.60	1	38	9.82	25.00	9.67
R$501.00 to R$1000.00	72	7	65	18.14	28.00	17.47	0	72	18.14	0.00	18.32
R$1001.00 to R$1500.00	125	6	119	31.49	24.00	31.99	3	122	31.49	75.00	31.04
R$1501.00 to R$2000.00	72	2	70	18.14	8.00	18.82	0	72	18.14	0.00	18.32
R$2001.00 to R$5000.00	73	3	70	18.39	12.00	18.82	0	73	18.39	0.00	18.58
More than R$5000.00	8	0	8	2.02	0.00	2.15	0	8	2.02	0.00	2.04
Not currently working	5	0	5	1.26	0.00	1.34	0	5	1.26	0.00	1.27
Don’t know the income	3	0	3	0.76	0.00	0.81	0	3	0.76	0.00	0.76
Total	397	25	372	100.00	100.00	100.00	4	393	100.00	100.00	100.00

*Relationship status*											
Married	79	3	76	19.90	12.00	20.43	0	79	19.90	0.00	20.10
Dating	13	2	11	3.27	8.00	2.96	0	13	3.27	0.00	3.31
Divorced	3	0	3	0.76	0.00	0.81	0	3	0.76	0.00	0.76
Living with a partner	206	10	196	51.89	40.00	52.69	2	204	51.89	50.00	51.91
Single	96	10	86	24.18	40.00	23.12	2	94	24.18	50.00	23.92
Total	397	25	372	100.00	100.00	100.00	4	393	100.00	100.00	100.00

*Sexual orientation*											
Homosexual	2	0	2	0.50	0.00	0.54	0	2	0.50	0.00	0.51
Heterosexual	380	23	357	95.72	92.00	95.97	4	376	95.72	100.00	95.67
Bisexual	15	2	13	3.78	8.00	3.49	0	15	3.78	0.00	3.82
Total	397	25	372	100.00	100.00	100.00	4	393	100.00	100.00	100.00

*Prenatal start*											
First 3 months	300	16	284	75.57	64.00	76.34	4	296	75.57	100.00	75.32
4–6 months	86	7	79	21.66	28.00	21.24	0	86	21.66	0.00	21.88
7–9 months	7	2	5	1.76	8.00	1.34	0	7	1.76	0.00	1.78
Don’t know	2	0	2	0.50	0.00	0.54	0	2	0.50	0.00	0.51
Did not start	2	0	2	0.50	0.00	0.54	0	2	0.50	0.00	0.51
Total	397	25	372	100.00	100.00	100.00	4	393	100.00	100.00	100.00

Most of the pregnant women had completed high school (51.51%), while 23.62% had completed elementary school or reported some high school education. Although women with lower educational levels (incomplete elementary school or less) tended to present higher proportions of HIV and syphilis positivity, these differences did not reach statistical significance (Tables 1 and 3). Regarding income, the largest proportion of participants reported monthly household incomes between R$1001 and R$1500 (31.91%), followed by the range of R$1501 to R$2000 (18.09%). A higher proportion of positive syphilis results was observed among pregnant women with monthly incomes below R$1000; however, this pattern represented a descriptive trend and was not statistically significant, and no independent association was confirmed in the analytical models (Tables 1 and 3).

Around half of the pregnant women lived with a partner (51.51%), and 24.37% were single. A trend of higher HIV/syphilis prevalence was observed among single pregnant women (Table 3). Approximately 75.63% began prenatal care in the first trimester, reflecting a relatively high adherence rate. However, the prevalence of infections appeared slightly higher among those who started prenatal care in the second or third trimester (Table 3).

Most pregnant women reported initiating sexual activity between the ages of 16 and 20 years (49.87%). A substantial proportion (45.34%) reported first sexual intercourse between the ages of 10 and 15 years, and this group showed a higher proportion of positive results for syphilis and HIV (76%); however, these differences represented descriptive trends and did not reach statistical significance (Tables 1 and 4). Similarly, pregnant women who initiated sexual activity after the age of 15 years tended to have a lower proportion of syphilis positivity, but this association was not statistically significant. Condom use was low overall, with 42.82% of pregnant women reporting never using condoms with regular partners. Among women reporting casual sexual partners, inconsistent condom use was frequent (Table 4).

**TABLE 4:** Sexual partnership characteristics and condom use according to syphilis and HIV serological status among study participants.

		Syphilis					HIV				
	Total	Positive	Negative	Total (%)	Positive (%)	Negative (%)	Positive	Negative	Total (%)	Positive (%)	Negative (%)
*Regular partner*											
Yes	369	22	347	92.95	88.00	93.28	4	365	92.95	100.00	92.88
No	25	2	23	6.30	8.00	6.18	0	25	6.30	0.00	6.36
I prefer not to answer	3	1	2	0.76	4.00	0.54	0	3	0.76	0.00	0.76
Total	397	25	372	100.00	100.00	100.00	4	393	100.00	100.00	100.00

*Condom use with a regular partner*											
Every time before attempting pregnancy	13	2	11	3.27	8.00	2.96	0	13	3.27	0.00	3.31
Never	170	6	164	42.82	24.00	44.09	0	170	42.82	0.00	43.26
Less than half the time	120	11	109	30.23	44.00	29.30	4	116	30.23	100.00	29.52
More than half the time	68	3	65	17.13	12.00	17.47	0	68	17.13	0.00	17.30
I prefer not to answer	26	3	23	6.55	12.00	6.18	0	26	6.55	0.00	6.62
Total	397	25	372	100.00	100.00	100.00	4	393	100.00	100.00	100.00

*Casual partner*											
Yes	64	6	58	16.12	24.00	15.59	0	64	16.12	0.00	16.28
No	328	18	310	82.62	72.00	83.33	4	324	82.62	100.00	82.44
I prefer not to answer	5	1	4	1.26	4.00	1.08	0	5	1.26	0.00	1.27
Total	397	25	372	100.00	100.00	100.00	4	393	100.00	100.00	100.00

*Number of casual partners in the last 12 months*											
One	38	5	33	59.38	83.33	56.90	0	38	59.38	0.00	59.38
Two	14	0	14	21.88	0.00	24.14	0	14	21.88	0.00	21.88
Three to five	11	1	10	17.19	16.67	17.24	0	11	17.19	0.00	17.19
More than 10 casual sexual partners	1	0	1	1.56	0.00	1.72	0	1	1.56	0.00	1.56
Total	64	6	58	100.00	100.00	100.00	0	64	100.00	0.00	100.00

*Condom use with casual partner*											
Every time	29	3	26	45.31	50.00	44.83	0	29	45.31	0.00	45.31
Never	13	2	11	20.31	33.33	18.97	0	13	20.31	0.00	20.31
Less than half the time	7	1	6	10.94	16.67	10.34	0	7	10.94	0.00	10.94
More than half the time	12	0	12	18.75	0.00	20.69	0	12	18.75	0.00	18.75
I prefer not to answer	3	0	3	4.69	0.00	5.17	0	3	4.69	0.00	4.69
Total	64	6	58	100.00	100.00	100.00	0	64	100.00	0.00	100.00

*Beginning of sexual life*											
10–15 years	180	19	161	45.34	76.00	43.28	1	179	45.34	25.00	45.55
16–20 years	198	5	193	49.87	20.00	51.88	3	195	49.87	75.00	49.62
21–25 years	15	1	14	3.78	4.00	3.76	0	15	3.78	0.00	3.82
> 26 years	4	0	4	1.01	0.00	1.08	0	4	1.01	0.00	1.02
Total	397	25	372	100.00	100.00	100.00	4	393	100.00	100.00	100.00

Approximately 11.81% of the pregnant women reported psychoactive substance use. This group presented a higher prevalence of syphilis positivity compared with nonusers (Table 5). In the adjusted analysis using Poisson regression with robust variance, psychoactive substance use remained significantly associated with syphilis positivity, with a prevalence ratio of 2.90 (95% CI: 1.28–6.56; *p* < 0.05), indicating nearly a threefold higher prevalence among users compared with nonusers (Tables 1 and 5). In contrast, 8.29% of pregnant women reported alcohol consumption. Although syphilis positivity was observed in this group, no statistically significant association was identified between alcohol use and syphilis infection in the bivariate analyses or in the final models (Tables 1 and 5).

**TABLE 5:** Psychoactive substance use and alcohol consumption according to syphilis and HIV serological status among the study participants.

	Syphilis						HIV					
	Total	Positive	Negative	Total (%)	Positive (%)	Negative (%)	Total	Positive	Negative	Total (%)	Positive (%)	Negative (%)
*Psychoactive substance use*												
Yes	47	7	40	11.84	28.00	10.75	47	1	46	11.84	25.00	11.70
No	350	18	332	88.16	72.00	89.25	350	3	347	88.16	75.00	88.30
Total	397	25	372	100.00	100.00	100.00	397	4	393	100.00	100.00	100.00

*Alcohol consumption*												
Yes	33	2	31	8.31	8.00	8.33	33	1	32	8.31	25.00	8.14
No	364	23	341	91.69	92.00	91.67	364	3	361	91.69	75.00	91.86
Total	397	25	372	100.00	100.00	100.00	397	4	393	100.00	100.00	100.00

Regarding syphilis awareness, 86.43% of pregnant women had heard of syphilis before the study, indicating a good level of exposure to the topic. Among the participants, 68.09% knew that syphilis could be transmitted from mother to child, while 72.86% had previously been tested for syphilis (Table 6). Regarding HIV knowledge, 96.23% of pregnant women had heard of HIV, indicating widespread awareness of this infection. Most pregnant women (91.71%) knew that HIV could be transmitted from mother to child, and a similar number understood HIV transmission in general. However, only 84.17% had been tested for HIV, indicating a gap between awareness and testing practices (Table 6).

**TABLE 6:** Knowledge and testing history for syphilis and HIV according to syphilis and HIV serological status among the study participants.

		Syphilis						HIV				
	Total	Positive	Negative	Total (%)	Positive (%)	Negative (%)	Total	Positive	Negative	Total (%)	Positive (%)	Negative (%)
*Have you ever heard about syphilis/HIV?*												
Yes	343	22	321	86.40	88.00	86.29	382	4	378	96.22	100.00	96.18
No	54	3	51	13.60	12.00	13.71	15	0	15	3.78	0.00	3.82
Total	397	25	372	100.00	100.00	100.00	397	4	393	100.00	100.00	100.00

*Do you know how syphilis/HIV can be transmitted?*												
Yes	249	19	230	62.72	76.00	61.83	364	3	361	91.69	75.00	91.86
No	148	6	142	37.28	24.00	38.17	33	1	32	8.31	25.00	8.14
Total	397	25	372	100.00	100.00	100.00	397	4	393	100.00	100.00	100.00

*Did you know that syphilis/HIV can be passed from mother to child?*												
Yes	270	20	250	68.01	80.00	67.20	364	3	361	91.69	75.00	91.86
No	127	5	122	31.99	20.00	32.80	33	1	32	8.31	25.00	8.14
Total	397	25	372	100.00	100.00	100.00	397	4	393	100.00	100.00	100.00

*Have you been tested for syphilis/HIV?*												
Yes	290	22	268	73.05	88.00	72.04	335	3	332	84.38	75.00	91.46
No	107	3	104	26.95	12.00	27.96	62	1	31	15.62	25.00	8.54
Total	397	25	372	100.00	100.00	100.00	397	4	363	100.00	100.00	100.00

## 4. Discussion

The presented results suggest a complex landscape of vulnerability to syphilis and HIV among pregnant women, with multiple interlinked factors, such as socioeconomic conditions, education, and risk behaviors. The relatively high prevalence of syphilis and HIV indicates significant exposure to these STIs, especially in low‐income populations with limited access to healthcare and fewer opportunities for preventive screening.

Recently, except for HIV, a general increase in STIs has been observed, even in highly developed countries [10, 11]. This increasing incidence rate of syphilis has also been observed in Brazil, rising from 8.9 per 1000 live births in 2014 to 35.4 per 1000 live births in 2024 [4]. A similar trend was seen in the state of Pará, where the incidence rate increased from 8.6 per 1000 live births to 30.2 per 1000 live births in 2024 [3]. According to the epidemiological bulletin on syphilis and HIV [3, 4], there was a significant decrease in reported cases in 2020 compared to previous years, followed by a substantial increase in 2021 and 2022. The decline in HIV and syphilis notifications observed in 2020 has been attributed to the impact of the COVID‐19 pandemic on healthcare systems and social behaviors. During this period, health services prioritized the response to COVID‐19, resulting in the interruption or reduction of nonessential services, including testing and treatment for sexually transmitted infections. Many healthcare facilities were partially redirected to COVID‐19 care, and testing and counseling services experienced temporary closures or reduced availability. Consequently, decreased access to diagnostic services led to a substantial reduction in testing, with a corresponding decline in the detection and reporting of new HIV and syphilis cases. A similar reduction in reported HIV and syphilis cases during 2020 was also documented by Sentís et al. [12], supporting the interpretation that this decline primarily reflects underdiagnosis and underreporting rather than a true reduction in transmission. In contrast, the detection rate of HIV among pregnant women remained stable during 2020 and 2021, while syphilis in pregnancy continued to show an increasing trend, indicating that, unlike other health services, prenatal care programs were not substantially disrupted during the pandemic period.

As observed in the epidemiological bulletins [3, 4], the COVID‐19 pandemic did not significantly disrupt prenatal healthcare services. These findings indicate that the prevalences identified in the present study are unlikely to be attributable to reduced access to prenatal care or diagnostic services during the pandemic. Instead, the 6.36% prevalence of syphilis and 1.01% prevalence of HIV observed in this study, which is consistent with the 5.2% prevalence of syphilis reported among pregnant women in Belém in 2019 and 2020 [9], suggest that similar prevalence levels reported in contemporaneous studies reflect the influence of persistent social, behavioral, and structural determinants rather than pandemic‐related service disruptions.

The present study identified psychoactive substance use as the main factor associated with syphilis positivity among pregnant women, remaining the only variable that showed a statistically significant association in the adjusted analyses. This finding is consistent with previous evidence from northern Brazil, including the study by Oliveira‐Filho et al. [13], which also reported a strong correlation between psychoactive substance use and HIV infection, reinforcing the role of substance use as a central marker of vulnerability to sexually transmitted infections in this region. Together, these findings suggest that drug use represents a convergent risk factor across different STIs and populations, including pregnant women receiving prenatal care.

The association between psychoactive substance use and syphilis likely reflects a complex interplay of behavioral, social, and structural factors, including reduced adherence to preventive practices, a higher likelihood of unprotected sexual activity, social marginalization, housing instability, poverty, and challenges in maintaining continuous engagement with healthcare services. In this context, psychoactive substance use may serve as a marker of multiple overlapping vulnerabilities that are not fully captured by conventional sociodemographic indicators. Therefore, its persistence in the multivariable model should not be interpreted as evidence of true independence from other social determinants, but rather as reflecting its close relationship with a broader constellation of interconnected risk factors. These findings should also be interpreted considering several limitations. Psychoactive substance use was self‐reported and may therefore be subject to social desirability bias, potentially resulting in underreporting and underestimation of its true prevalence. In addition, information regarding the route or mode of psychoactive substance use was not collected. Different routes of administration may be associated with distinct behavioral and epidemiological risk profiles, and the absence of these data may have limited a more nuanced assessment of the relationship between substance use and syphilis. From a public health perspective, these findings highlight the importance of integrating substance use screening, targeted counseling, and harm‐reduction strategies into prenatal care. Addressing psychoactive substance use during pregnancy may contribute to reducing syphilis transmission and support efforts to eliminate vertical transmission, particularly in settings characterized by persistent social and structural inequalities.

Although other sociodemographic and behavioral variables, such as lower household income, early sexual initiation, lower educational attainment, and inconsistent condom use, did not reach statistical significance in the final models, their descriptive trends indicate that these factors may contribute indirectly to increased vulnerability. Among the evaluated variables, women with monthly household incomes above R$1000 showed a lower proportion of syphilis positivity compared with those earning below this threshold. This finding remains relevant considering the socioeconomic profile observed in the study population, in which most pregnant women reported low purchasing power, with 59.55% living on up to R$1500 per month and 27.64% earning less than the minimum wage in 2021 (R$1100). The overall socioeconomic profile observed in the study points to limited purchasing power and social vulnerability, which are widely recognized as contextual factors that may hinder timely access to healthcare services, preventive examinations, and continuity of prenatal follow‐up [14]. In this sense, income appears to operate less as an isolated determinant and more as a structural condition that interacts with other social and behavioral dimensions of vulnerability. Furthermore, the study population was restricted to pregnant women who accessed prenatal care services, which may have resulted in an underestimation of the true burden of syphilis and HIV in the target population. Women not engaged in prenatal care may experience greater social vulnerability and barriers to healthcare access, potentially leading to different infection profiles. Unfortunately, information on the proportion of pregnant women not accessing prenatal care was not available for the study population. Similarly, although ethnic self‐identification and educational attainment were not independently associated with infection outcomes, the distribution of these variables highlights potential inequalities related to access to education and health care. Lower levels of schooling may affect health literacy and autonomy in sexual and reproductive decision‐making. Behavioral variables followed a comparable pattern. Early sexual initiation, inconsistent condom use, and alcohol consumption did not show statistically significant associations, but collectively delineated a context of increased exposure to risk. Notably, the discrepancy between reported knowledge of sexually transmitted infections and the adoption of preventive practices suggests that information alone is insufficient to promote behavioral change. These findings indicate that vulnerability is likely cumulative and multifactorial, emerging from the interaction between social conditions, behavioral patterns, and access to supportive services.

Taken together, these trends suggest that the observed prevalence of HIV and syphilis among pregnant women reflects broader social and structural determinants rather than the effect of single risk factors. Although Brazil has implemented comprehensive public policies addressing sexual and reproductive health [15], the patterns identified in this study underscore the need to strengthen integrated approaches that combine healthcare delivery with education, social support, and equity‐oriented interventions. Such strategies are essential to reduce persistent vulnerabilities and to advance toward the elimination of vertical transmission of syphilis and HIV, particularly among socially disadvantaged populations.

## 5. Conclusion

This study provides evidence that, among the sociodemographic and behavioral factors evaluated, psychoactive substance use emerged as the main variable independently associated with syphilis positivity among pregnant women receiving prenatal care in Belém, Pará. While other factors, such as low income, early sexual initiation, limited educational attainment, and inconsistent condom use, showed descriptive trends toward higher vulnerability, these associations did not reach statistical significance in the final analytical models, suggesting that their effects may be mediated by broader social and structural contexts.

The findings indicate that the prevalence of syphilis and HIV observed in this population is consistent with contemporaneous studies and national surveillance data, reinforcing that prenatal care services remained functional during the study period and that the identified prevalence levels are unlikely to be explained by disruptions in healthcare access. Instead, the results highlight the role of persistent social vulnerability and risk behaviors, particularly substance use, as key drivers of infection risk during pregnancy.

From a public health perspective, these results underscore the importance of strengthening prenatal care strategies that integrate screening for psychoactive substance use, targeted counseling, harm‐reduction approaches, and linkage to psychosocial support services. Addressing substance use during pregnancy may represent a critical opportunity to reduce syphilis transmission and to advance toward the elimination of vertical transmission of sexually transmitted infections. Future studies should further explore the complex interactions between social determinants, behavioral factors, and healthcare access to inform more effective and equitable maternal and child health interventions.

## Funding

This study was funded through Letter Agreement TC 66–SCON2019‐00400 between the Pan American Health Organization (PAHO/WHO) and the Department of Chronic Diseases and Sexually Transmitted Infections—DCCI/SVS/MS.

## Ethics Statement

The project received approval from the Brazilian Ministry of Health—National Health Council—National Research Ethics Commission (No. 19146919.3.0000.0019) and da Organización Panamericana de la Salud Comité de Revisión Ética (PAHOERC‐2019‐08‐0059). All participants who met the study criteria signed an informed consent form (ICF) for the Evandro Chagas Institute Biobank.

## Consent

The authors have nothing to report.

## Conflicts of Interest

The authors declare no conflicts of interest.

## Data Availability

The data that support the findings of this study are available upon request from the corresponding author. The data are not publicly available due to privacy or ethical restrictions.
